# Supplementary material for: Specific Binding Ratio Estimation of [123I]-FP-CIT SPECT Using Frontal Projection Image and Machine Learning
Source: Diagnostics (Basel). 2023 Apr 7;13(8):1371. doi: 10.3390/diagnostics13081371 (PMC10137239; doi:10.3390/diagnostics13081371)
Supplement: Supplementary file 1 [file diagnostics-13-01371-s001.zip › diagnostics-2243606-supplementary.pdf]

diagnostics

MDPI

Article

Supplementary Materials: Specific Binding Ratio Estimation of [^{123}I]-FP-CIT SPECT Using Frontal Projection Image and Machine Learning

Akinobu Kita [1,*], Hidehiko Okazawa [2,*], Katsuya Sugimoto [1], Nobuyuki Kosaka [3], Eiji Kidoya [1] and Tetsuya Tsujikawa [3]

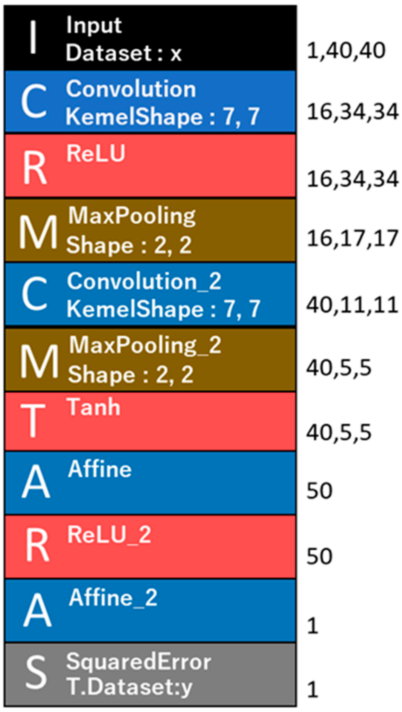

(**a**)

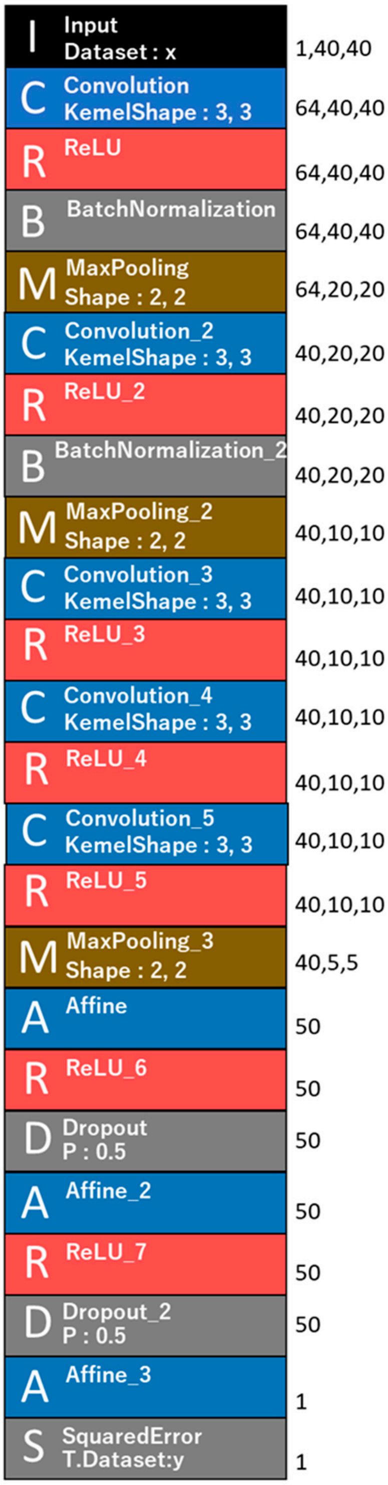

(**b**)

Figure S1. Two types of network diagrams, (**a**) LeNet and (**b**) AlexNet. The layer's function is indicated by square boxes. The specifications for each layer are depicted in the figures on the right side of the box. The web site https://dl.sony.com/ (accessed on 6 April 2023) describes additional details in this figure (Sony Network Communications Inc., 2020).
